# Opposite Distortions in Interval Timing Perception for Visual and Auditory Stimuli with Temporal Modulations

**DOI:** 10.1371/journal.pone.0135646

**Published:** 2015-08-20

**Authors:** Kenichi Yuasa, Yuko Yotsumoto

**Affiliations:** Department of Life Sciences, The University of Tokyo, Tokyo, Japan; Duke University, UNITED STATES

## Abstract

When an object is presented visually and moves or flickers, the perception of its duration tends to be overestimated. Such an overestimation is called *time dilation*. Perceived time can also be distorted when a stimulus is presented aurally as an auditory flutter, but the mechanisms and their relationship to visual processing remains unclear. In the present study, we measured interval timing perception while modulating the temporal characteristics of visual and auditory stimuli, and investigated whether the interval times of visually and aurally presented objects shared a common mechanism. In these experiments, participants compared the durations of flickering or fluttering stimuli to standard stimuli, which were presented continuously. Perceived durations for auditory flutters were underestimated, while perceived durations of visual flickers were overestimated. When auditory flutters and visual flickers were presented simultaneously, these distortion effects were cancelled out. When auditory flutters were presented with a constantly presented visual stimulus, the interval timing perception of the visual stimulus was affected by the auditory flutters. These results indicate that interval timing perception is governed by independent mechanisms for visual and auditory processing, and that there are some interactions between the two processing systems.

## Introduction

At almost every moment in life, we consciously and unconsciously perceive durations of events. These durations can range from several hundred milliseconds to hours, or even days. The perception of durations lasting from milliseconds to seconds is called interval timing perception, which has been widely studied using various modalities [1–3]. These perceived durations can be distorted by temporal modulations of sensory stimuli. For example, if a visually presented stimulus moves, expands, or flickers, its perceived duration tends to be overestimated [4–7].

Interval timing perception can be explained by an internal pacemaker system. There are some pacemakers, which emit a series of internal clock pulses at a particular frequency, and these accumulated pulses give the perception of time [8–10]. Treisman, Faulkner [11] demonstrated that the perceived durations of flutters varied periodically depending on the fluttering frequencies, and explained these results with the internal clock model. Such time distortion was interpreted to be a result of the rapid intermittent modulations in the stimuli, which affected the frequency of the internal pacemaker [12–16]. The internal clock model was developed using populations of coincidental neural activations. This so-called Striatal Beat Frequency (SBF) model hypothesized that oscillator neurons projected onto striatal spiny neurons [4, 16, 17], and successfully explained the relationship between interval timing perceptions and neural oscillatory activities.

Distortions of perceived durations have been reported using stimuli of many different modalities, including auditory flutters [11–14, 18–21], visual flickers [5, 7, 15, 22–26], and tactile vibrations [27, 28]. In these studies, rapid intermittent stimuli (e.g., flickers or flutters) were presented during time estimation tasks, and their perceived durations were measured in various ways. In some cases, the intermittent stimuli themselves were the target stimuli for the duration estimation, while in others, the intermittent stimuli were presented before or after the target stimuli. Auditory flutters, visual flickers, and tactile vibrations usually induced an overestimation of their durations. The neural mechanisms of those time distortions were interpreted within the framework of the internal clock model [12–16]. Using this theory, the overestimation was interpreted as pacemaker acceleration. When temporal modulation of the external stimulus accelerated the internal pacemaker clock, the emitted pulses for the specified duration increased, such that subjective time became dilated. Similarly, duration underestimation was interpreted as pacemaker deceleration.

Although the pacemaker theory can be used to explain the general mechanism of time distortion regardless of stimuli modality, the characteristics of temporal processing between auditory and visual modalities are different. The visual modality dominates the auditory modality with regard to spatial information [29, 30], while the auditory modality dominates the visual modality with regard to temporal information [31–33]. These interactions are attributed to the higher and more accurate temporal resolution associated with the auditory modality [34–36].

Differences and interactions between visual and auditory modalities have also been found in time perception. When temporal information is conflicted between these modalities, auditory stimuli can affect the perception of visually presented stimuli [37–42]. For example, an auditory oddball stimulus dilated the duration of a visually presented stimulus, while a visual oddball stimulus did not affect the perceived duration of an auditory stimulus [43]. In addition, stimulating the auditory cortex with transcranial magnetic stimulation (TMS) affected the temporal processing of a visual stimulus, while stimulating the visual cortex did not affect the temporal processing of an auditory stimulus [44]. Furthermore, durations of auditory stimuli tended to be perceived as longer than the same durations of visual stimuli [4, 45–51].

Interpreted within the framework of the internal clock model, the differences between auditory and visual modalities could be attributed to differences in the clock rates of their pacemakers [6, 49–52]. The interactions between auditory and visual modalities suggest that they recruit different accumulation systems of internal clock pulses. Such multiple accumulators with different modalities were also predicted in the context of parallel timing processing [53, 54]. However, there have not been any *direct* comparisons between auditory and visual stimuli to determine how temporal modulations of the stimulus can affect duration perception. In fact, as stated earlier, most studies that applied temporal modulations to the stimulus reported time dilation with both auditory and visual stimuli.

Recently, we reported frequency-specific modulations in perceived durations using flickering visual stimuli with various temporal frequencies, and proposed a model to explain time dilation using neural entrainment induced by an external visual flicker [55]. In the proposed model, neural entrainments are induced by visual flickers in a frequency-specific manner. The neural entrainments modulate base temporal frequencies of the internal clock, resulting in time dilation. If the internal clock operates with a general base temporal frequency for different modalities, neural entrainment would induce similar distortions in perceived durations regardless of the modality. On the other hand, if the base temporal frequencies of the internal clock are modality-specific, the same temporal modulation could induce modality-dependent changes in perceived durations. In the present study, we modulated the temporal frequencies of an auditory stimulus and a visual stimulus with the same temporal frequency of 10.9 Hz, and measured the resulting time perception. By comparing the effects of temporal modulation on the perceived time for an auditory modality and for a visual modality, we investigated modality specificity and the between-modality interactions of interval timing perception.

Perception of interval timing for sub-second and supra-second stimuli is influenced by different neural mechanisms [2, 56–64]. Sub-second time perception tended to employ a relatively automatic process, while supra-second time perception requires cognitive processing [59, 60]. The extended SBF model with an integrated working memory component (SBFm) successfully demonstrated the existence of these different mechanisms [64, 65].

In this study, we examined interval timing perception using 1-s and 3-s stimuli in order to discuss the roles of temporal frequencies, as well as interpret our results within the framework of peri-second and supra-second interval timing perception.

## Experiment 1: Duration Comparisons with Auditory Flutters

In Experiment 1, we evaluated the perceived durations of auditory stimuli using continuously presented auditory tones and fluttering auditory tones.

### Experiment 1:Methods

#### Participants

Nine volunteers (4 male, mean age = 20.4 years [range: 18–27 years]) participated in Experiment 1. All had normal hearing and normal or corrected-to-normal vision. Participants were unaware of the purpose of the experiment, and gave written informed consent for their participation in the experiment. The protocol was approved by the institutional review boards at the University of Tokyo.

#### Apparatus

The auditory stimuli were presented through an Audio Stream Input Output (ASIO) compliant USB digital-to-analog converter (DS-DAC-10, KORG INC.) and ER-2 insert-tube-earphones (Etymotic Research). Participants sat in a dark soundproof room and used a chin support placed 57.3 cm in front of a computer monitor. A computer keyboard was placed between participants and the monitor to register participants’ responses. A workstation (Windows 7 Professional 64bit) controlled stimulus presentations and recorded the participants’ responses. Instructions and visual fixations were displayed on a 23.6-inch VIEWPixx/3D lite monitor (VPixx Technologies) with 1920 × 1080 resolution at a refresh rate of 120 Hz.

#### Stimuli and procedure

**Fig 1:**
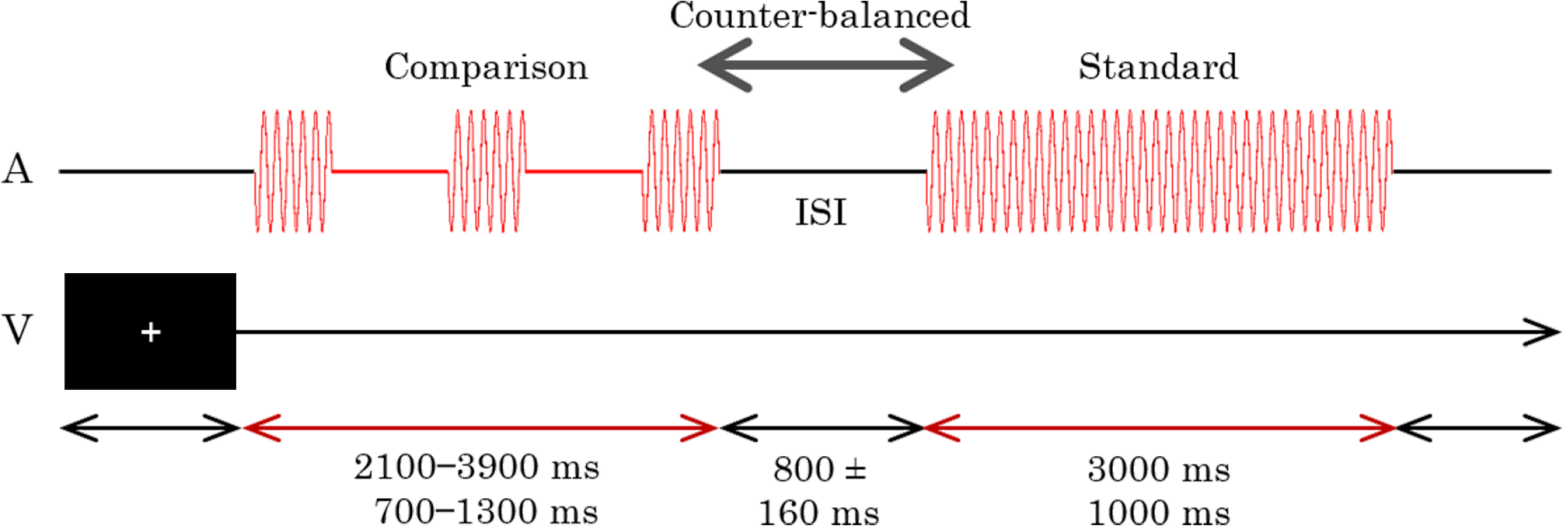
Time course of Experiment 1. Only auditory stimuli (A) were presented in this experiment. The comparison stimuli were presented continuously in half of the trials and intermittently as a flutter in the remaining trials. A visual fixation cross (V) was displayed throughout the trial. The stimulus durations and ISI (inter-stimulus-interval) were varied in each trial.

The stimulus configurations are shown in Fig 1. Each trial consisted of sequential presentations of a standard stimulus and a comparison auditory stimulus. The standard stimulus was always presented continuously, while the comparison stimulus was presented either continuously or intermittently. In Experiment 1, the continuous stimulus was a simple tone (1,000 Hz, 80 dB), and the intermittent stimulus was a 10.9-Hz flutter (1,000 Hz, 80 dB). The flutter consisted of a series of simple tones, which lasted for 16.7 ms, including a rise and a fall time of about 1 ms each, and silences for 75.0 ms; they were sampled at 44.1 kHz and quantized to 16 bits. Auditory stimuli were generated by the MATLAB software (Mathworks, R2013b) using the Psychophysics Toolbox extensions [66–68]. Sound levels were calibrated with a WS1361 sound level meter (Wensn). The order of the standard and comparison stimuli was counterbalanced across trials.

Standard stimuli had fixed durations of either 3-s or 1-s, while comparison stimuli had seven durations, such that each duration differed by 0%, ±10%, ±20%, or ±30%. No sound was presented during the inter-stimulus interval (ISI), which varied randomly between 640–960 ms. A white fixation cross was visually presented on the monitor throughout the session.

Participants were instructed to attend to the auditory stimuli without counting their durations and to visually fixate on the white fixation cross during the task. Participants were asked to determine whether the presentation of the second stimulus was longer than that of the first stimulus by pressing a corresponding key after each trial. There was no time limit for the response. Each condition (7 comparison durations × 2 types of comparison stimuli [continuous or flutter]) was repeated 40 times for each standard duration (3-s or 1-s). Therefore, each participant completed 1,120 trials over a 4-day period.

### Experiment 1:Results and Discussion

**Fig 2:**
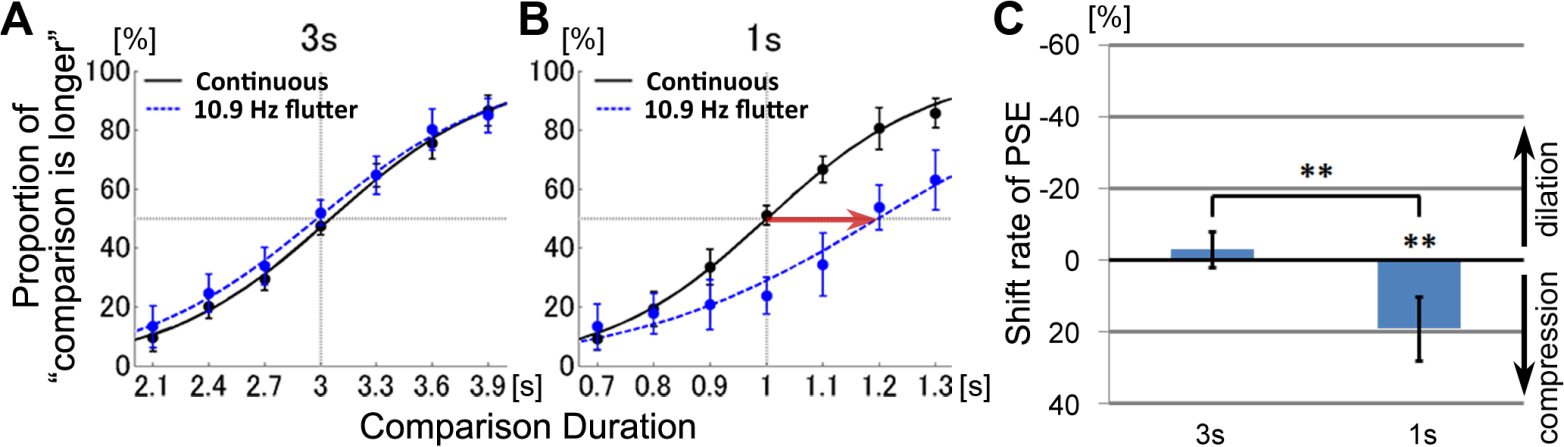
Experiment 1. Effect of auditory flutters on interval timing perception. Psychometric functions showed the mean probability of the comparison stimulus perceived longer than the standard stimulus. Results for the standard duration of 3-s (A) and 1-s (B) were plotted separately. The red arrow indicates a significant PSE shift. (C) The shift rates of the PSE (point of subjective equality). Error bars represent the 95% confidence interval for each comparison duration.

The proportions that the comparison stimulus was perceived longer than the standard stimulus are plotted in Fig 2A and 2B. Data were averaged across subjects and fitted to logistic psychometric functions. When the standard auditory stimulus was presented for 3-s, the curves for the continuous auditory tone and for the fluttering tones overlapped (Fig 2A). In contrast, when the standard auditory stimulus was presented for 1-s, the fluttering stimuli tended to be perceived as shorter than the non-fluttering stimuli (Fig 2B).

The magnitude of distortion of the perceived duration was defined as the shift in point of subjective equality (PSE), where psychometric functions revealed that 50% proportions of the comparison stimulus were perceived to be longer. If the perceived duration were distorted, the PSE obtained by the flutter stimuli would shift from that obtained by the continuous stimuli, which in theory was equal to the actual durations of the standard stimulus (3-s or 1-s). In order to normalize the shift amount of PSE, we divided the amount of shift duration by the standard duration, and obtained the shift rate of PSE. Negative values of the shift rate indicate that the comparison stimulus of same duration was perceived as longer, i.e. time dilation. Positive values indicate that the comparison stimulus of same duration was perceived as shorter, i.e. time compression. The shift rates of the average PSEs were −2.98% ± 6.54% (SD) for the 3-s standard duration, and 19.20% ± 11.68% (SD) for the 1-s standard duration (Fig 2C). As shown in Fig 2C, the PSE shift rate was significantly larger than zero for the 1-s duration (t(8) = 4.93, *p* = 0.001, *d* = 1.64), but not the 3-s duration (t(8) = 1.39, *p* = 0.20, *d* = 0.46). The shift rates for the 3-s and 1-s durations were significantly different (t(8) = 6.00, *p <* 0.001). These results indicate that perceived time was compressed by the 1-s auditory flutter. However, such compression was not observed with the 3-s duration.

Based on previous studies [11–14, 18–21], we predicted that the durations of fluttering stimuli would be perceived as longer than the durations of continuous stimuli. However, the results in Experiment 1 contradict our prediction, because we observed time compression with the 1-s fluttering stimulus. Majority of previous studies that reported time dilation with auditory flutters presented the fluttering stimuli separate from the target stimulus. Typically, a fluttering stimulus was first presented for 3 to 5 s, and subsequently, a target stimulus was presented, rather than a fluttering target stimulus. In those studies, fluttering stimuli preceded target stimuli, and the perceived durations dilated for both the sub-second stimuli [13, 14, 19, 21] and for the supra-second stimuli [14, 18, 19]. In contrast, compression of the perceived duration was reported when the auditory flutter followed the sub-second target intervals [13]. Some studies presented fluttering stimuli as the target stimuli, as we did in this experiment, and reported time dilations [11, 13, 20]. These conflicting results (time dilation versus time compression induced by auditory flutter) may be attributed to differences in experimental tasks and procedures [69–72]. Treisman, Faulkner (11) used a verbal estimation task, in which the participants were asked to report the estimated duration using their words. Burle and Casini (20) used a reproduction task, in which participants pressed a key for the same duration that they perceived fluttering stimuli. These two tasks did not require comparisons between two durations. In addition, Treisman, Faulkner (11) continuously displayed an asterisk with the auditory flutter, while Burle and Casini (20) displayed a blue LED, which was lit during reproducing. This was in contrast with our experiment, where no simultaneous visual stimulus was presented. Because these studies did not give specific instructions regarding visual stimuli, the presence of the visual stimuli may have affected the estimation of stimuli durations. Ono and Kitazawa (13) used a pair comparison task, in which two auditory stimuli were presented sequentially, and participants reported which stimulus was perceived to be longer. This experimental task was similar to ours; however, the auditory flutters used in their study were 5 Hz and 25 Hz flutters, with no control condition presenting continuous auditory tones. Therefore, we cannot directly compare their results with ours.

In Experiment 1, time distortion was observed only with the 1-s duration, and not the 3-s duration. These results support the idea that there are different systems of interval timing perception for sub-second and supra-second durations. In Experiment 2, we conducted a similar experiment using visual stimuli.

## Experiment 2: Duration Comparisons of Visual Flickers

In Experiment 2, the perceived durations of visual stimuli were examined using continuously presented visual stimuli and flickering visual stimuli.

### Experiment 2: Methods

#### Participants

Ten volunteers (5 male, mean age = 20.2 years [range: 18–27 years]) participated in Experiment 2. Two of the subjects also participated in Experiment 1. All had normal or corrected-to-normal vision and hearing. All participants were unaware of the purpose of the experiment, and gave written informed consent for their participation in the experimental protocol, which was approved by the institutional review boards at the University of Tokyo.

#### Apparatus

All visual stimuli were displayed on a VIEWPixx/3D lite monitor. In Experiment 2, participants did not wear earphones. Other components of the experimental apparatus were the same as those described in Experiment 1.

#### Stimuli and procedure

The procedure in Experiment 2 was similar to Experiment 1 except for the following differences. Instead of auditory stimuli, white Gaussian blobs were presented on the monitor. The standard stimulus was a continuously presented Gaussian blob (SD = 1.45°, 50.3 cd/m^2^), while the comparison stimulus was either a continuously presented Gaussian blob or a Gaussian blob presented with 10.9-Hz flickers. The flickers consisted of white Gaussian blobs for 2 video frames (16.7 ms) and blanks for 9 video frames (75.0 ms). Visual stimuli were generated by the MATLAB (Mathworks) software with Psychtoolbox. Luminescence was calibrated with a ColorCal MKII Colorimeter (Cambridge Research Systems).

**Fig 3:**
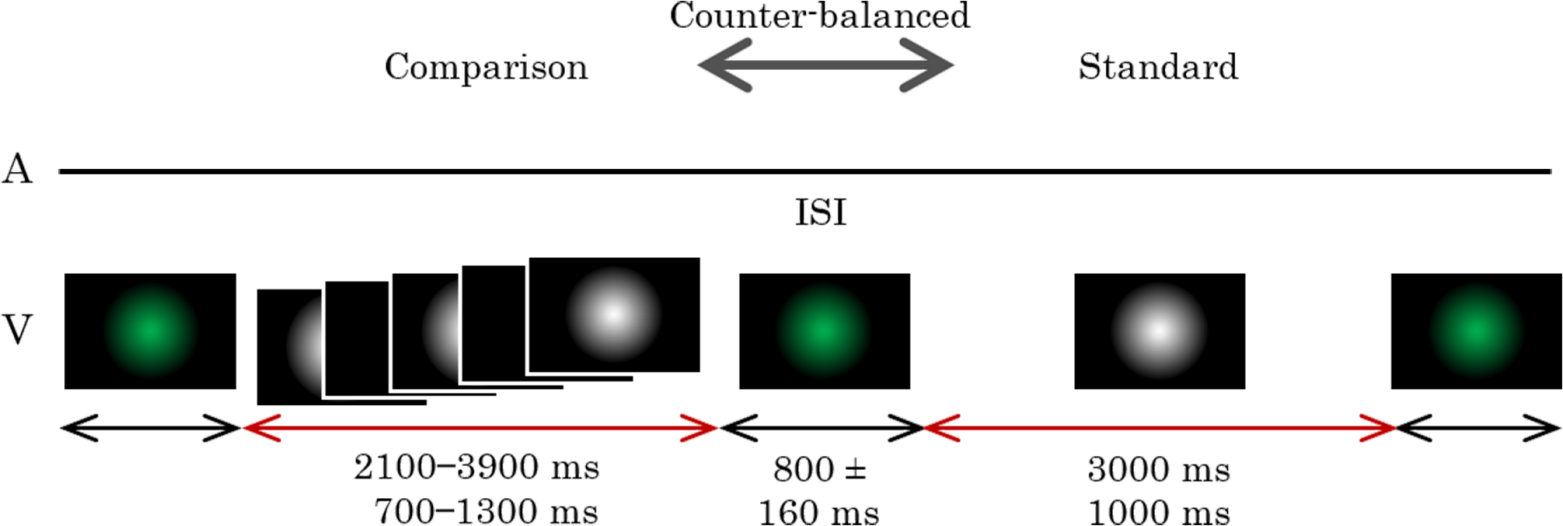
Time course of Experiment 2. Only visual stimuli (V) were presented in this experiment. The comparison stimuli were presented continuously in half of the trials and intermittently as a flicker in the remaining trials. No auditory stimulus (A) was presented throughout the trial. The stimulus durations and ISI (inter-stimulus-interval) were varied in each trial.

Standard stimuli durations were fixed to either 3-s or 1-s. Comparison stimuli had seven durations, with each standard duration differing by 0%, ±10%, ±20%, or ±30%. Green Gaussian blobs (SD = 1.45°) were presented before the stimulus presentation, during the ISI, and after the stimulus presentation to avoid afterimages. The green Gaussian blobs were adjusted to equal luminesce as the white Gaussian blobs. The stimulus configurations are shown in Fig 3.

Participants were instructed to observe the visual stimuli without counting their durations. Participants were instructed to respond whether the second stimulus had a longer duration than the first stimulus by pressing a corresponding key after each trial. There was no time limit for the response. Each condition (7 comparison durations × 2 types of comparison stimuli [continuous or flicker]) was repeated 40 times for each standard duration (3-s or 1-s), and each participant completed 1,120 trials over a 4-day period.

### Experiment 2: Results and Discussion

One participant was excluded from the analyses because his response did not exceed chance, even when the comparison durations differed from the standard durations by ±30%.

**Fig 4:**
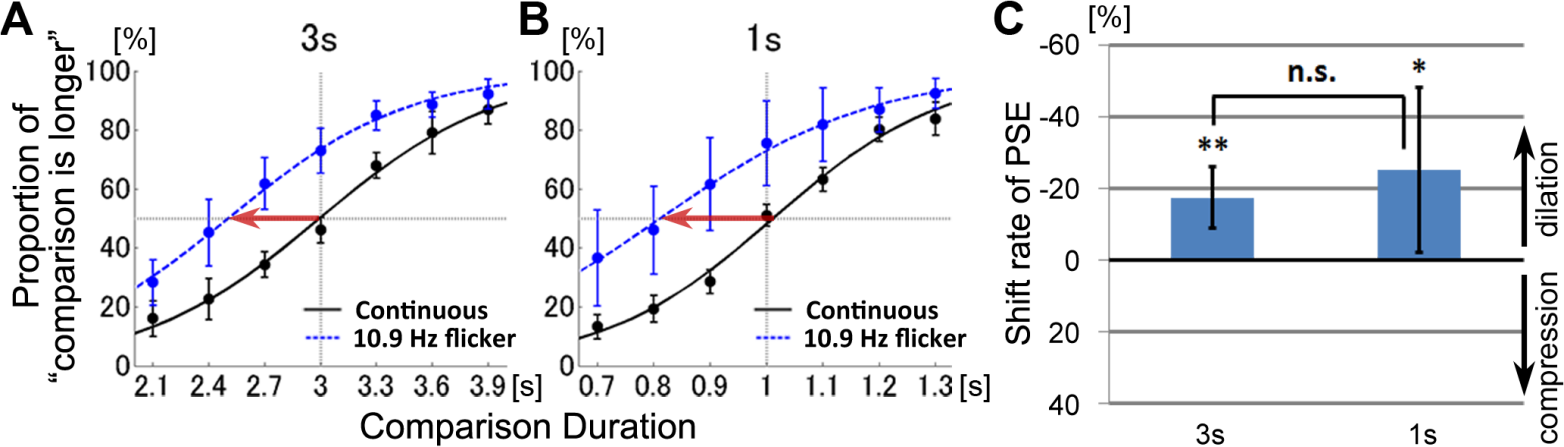
Experiment 2. Effects of visual flickers on interval timing perception. Psychometric functions showed the mean probability of the comparison stimulus perceived longer than the standard stimulus. The results for the standard duration of 3-s (A) and 1-s (B) were plotted separately. The red arrows indicate significant PSE shifts. (C) The shift rates of the PSE (point of subjective equality). Error bars represent the 95% confidence interval for each comparison duration.

The proportions that the comparison stimulus was perceived longer than the standard stimulus are plotted in Fig 4A and 4B. Data were averaged across subjects and fitted to logistic psychometric functions. The curves for flickering visual stimuli shifted towards the left compared to curves for continuously presented visual stimuli (Fig 4A and 4B). These data indicate that durations of flickering stimuli were perceived to be longer than durations of non-flickering stimuli, for both the 3-s and 1-s durations. These results indicate a tendency to overestimate flicking visual stimuli duration, which is consistent with previous studies [5, 7, 22–26]. The shift rates of the average PSEs were −17.27% ± 11.16% (SD) for the 3-s duration, and −25.15% ± 29.83% (SD) for the 1-s duration (Fig 4C). The standard deviation was large for the 1-s duration, which was due to one outlier. The PSE shift rates were significantly smaller than zero for the 3-s duration (t(8) = 4.95, *p* = 0.001, *d* = 1.55) and for the 1-s duration (t(8) = 2.60, *p* = 0.04, *d* = 0.84). There was no significant difference between the shift rates for the 3-s and 1-s conditions (t(8) = 1.24, *p* = 0.26).

The PSE shifts were smaller than zero, indicating that the flickering visual stimuli induced time dilation. Unlike Experiment 1, the PSE shift directions in Experiment 2 were consistent between the 3-s and 1-s durations, indicating that visual flickers induced time dilation for both the peri-second and the supra-second stimuli.

## Experiment 3: Duration Comparisons of Simultaneously Presented Auditory Flutters and Visual Flickers

When only one modality was used for interval timing perception (Experiments 1 and 2), the auditory and visual modalities exhibited opposite characteristics with regard to distortions of time perception (i.e., compression with auditory flutters, and dilation with visual flickers). These results led to the next question: what would happen to time perception when auditory and visual stimuli were presented simultaneously? Experiment 3 examined the perceived durations for combined audio-visual stimuli, using simultaneously presented auditory and visual stimuli.

### Experiment 3: Methods

#### Participants

Nine volunteers (4 male, mean age = 22 years [range: 18–32 years]) participated in Experiment 3. Two of the subjects also participated in Experiment 1 and 2. All had normal or corrected-to-normal vision and hearing. All participants were unaware of the purpose of the experiment, and gave written informed consent for their participation in the experimental protocol, which was approved by the institutional review boards at the University of Tokyo.

#### Apparatus

All visual stimuli were displayed on the VIEWPixx/3D lite monitor. Other components of the experimental apparatus were previously described in the Methods sections of Experiment 1 and 2.

#### Stimuli and procedure

**Fig 5:**
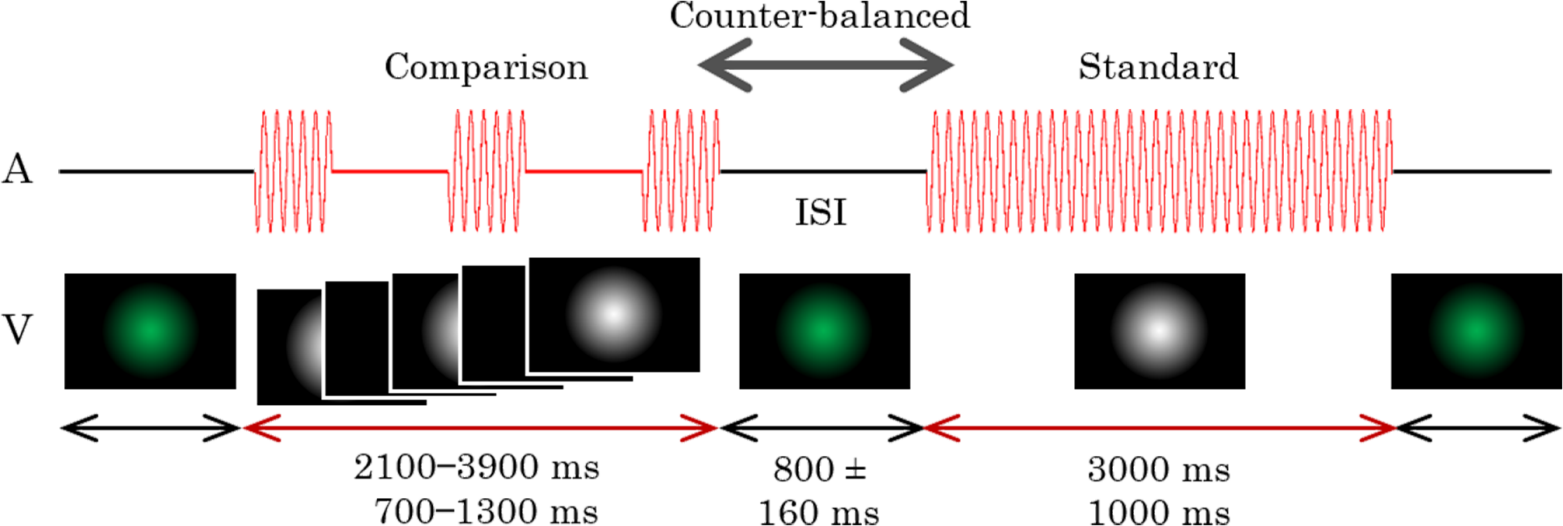
Time course of Experiment 3. Both auditory (A) and visual (V) stimuli were presented simultaneously. The comparison stimuli fluttered and flickered in half of the trials, and their durations varied from those of the standard stimuli. The stimulus durations and ISI (inter-stimulus-interval) were varied in each trial.

The procedure in Experiment 3 was similar to those described in Experiment 1 and 2. The stimulus configurations are shown in Fig 5. The auditory and visual stimuli were presented simultaneously for each standard and comparison stimulus. The standard stimuli were a simple tone (1,000 Hz, 80 dB) presented with a stationary Gaussian blob (SD = 1.45°, 50.3 cd/m^2^), while the comparison stimuli were either the same as the standard stimuli or a 10.9 Hz synchronized flutter presented with a 10.9 Hz flickering Gaussian blob.

The standard duration was either 3-s or 1-s, while the comparison duration was one of seven durations (different by 0%, ±10%, ±20%, or ±30% from the standard duration). Equiluminant green Gaussian blobs (SD = 1.45°) and silences were presented before the stimulus presentation, during the ISI, and after the stimulus presentation. The auditory and visual stimuli were always in synchrony, such that auditory flutters were always presented with visual flickers at the same temporal frequency, while the continuous auditory tone was always presented with the continuous visual blob. Durations of the simultaneously presented auditory and visual stimuli were always the same.

Participants were instructed to observe the auditory and visual stimuli without counting their durations, and to respond whether the duration of the second stimulus was longer than the first stimulus by pressing a corresponding key after each trial. There was no time limit for the response. Each condition (7 comparison durations × 2 types of comparison stimuli [continuous or flutter/flicker]) was repeated 40 times for each standard duration (3-s or 1-s), and each participant completed 1,120 trials over a 4 day period.

### Experiment 3: Results and Discussion

**Fig 6:**
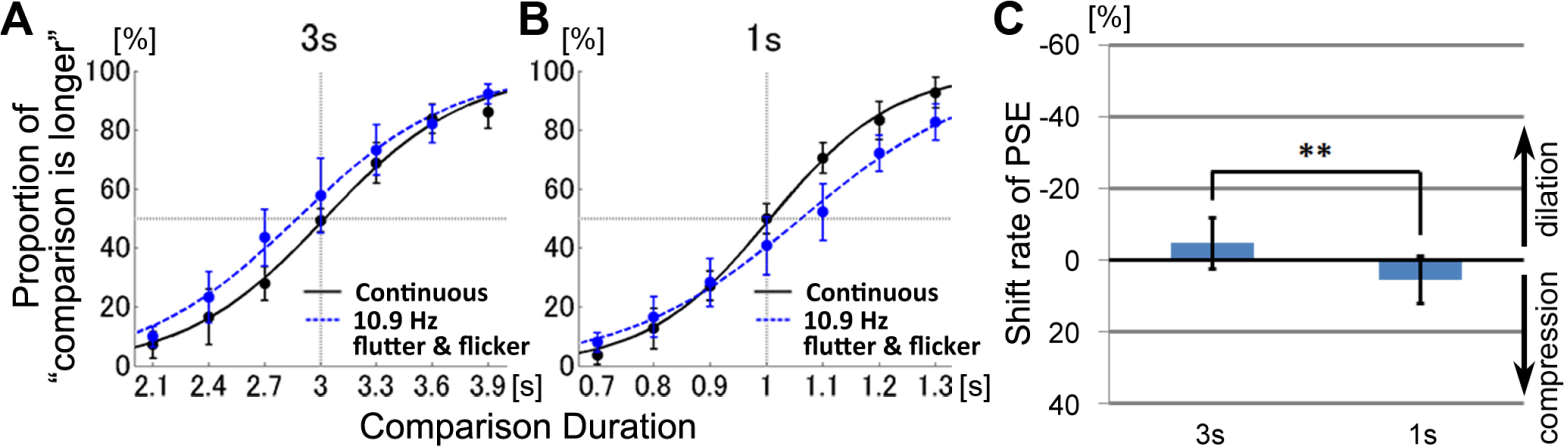
Experiment 3. Effects of simultaneously presented flutters/flickers on interval timing perception. Psychometric functions showed the mean probability of the comparison stimulus perceived longer than the standard stimulus. The results for the standard duration of 3-s (A) and 1-s (B) were plotted separately. (C) The PSE (point of subjective equality) shift rates between the continuous audio-visual stimuli and the simultaneously fluttering tones and flickering blobs. Error bars represent the 95% confidence interval for each comparison duration.

The proportions that the comparison stimulus was perceived longer than the standard stimulus are plotted in Fig 6A and 6B. Data were averaged across subjects and fitted to logistic psychometric functions. For both the 3-s and 1-s durations, the curves for the continuous stimuli and for flickering/fluttering stimuli overlapped (Fig 6A and 6B), indicating that the simultaneous flutters and flickers did not induce distortions of the perceived durations for either the 3-s or 1-s condition. The shift rates of the average PSEs were −4.67% ± 9.35% (SD) for the 3-s duration, and 5.52% ± 8.63% (SD) for the 1-s duration (Fig 6C). PSE shift rates were not significantly different from zero for the 3-s duration (t(8) = 1.50, *p* = 0.17, *d* = 0.50) or for the 1-s duration (t(8) = 1.92, *p* = 0.09, *d* = 0.64). The shift rates between the 3-s and the 1-s conditions, however, were significant (t(8) = 4.00, *p* = 0.004).

The simultaneously presented audio-visual stimuli did not induce perceived time distortions. The fluttering auditory stimulus induced time compression in Experiment 1, and the flickering visual stimulus induced time dilation in Experiment 2. No distortion was observed with the audio-visual flutter/flicker stimuli in this experiment, which can be interpreted as a cancellation of the conflicting time distortion effects.

## Experiment 4: Duration Comparisons of Auditory Flutters and Continuously Presented Visual Stimuli

In Experiment 3, we showed that simultaneously presented flutters/flickers induced neither compression nor dilation, indicating an interaction between these modalities. To further investigate these interactions, we presented auditory flutters with non-flickering visual stimuli and measured perceived time while the subjects were instructed to ignore the auditory stimulus. In Experiment 4, we sought to examine whether the ignored auditory stimulus could still affect perception of visually presented information.

### Experiment 4: Methods

#### Participants

Eight volunteers (6 male, mean age = 20.3 years, (range: 18–27 years) participated in Experiment 4. One of the subjects also participated in Experiment 1, 2 and 3. All had normal or corrected-to-normal vision and hearing. All participants were unaware of the purpose of the experiment, and gave written informed consent for their participation in the experimental protocol, which was approved by the institutional review boards at the University of Tokyo.

#### Apparatus

All components of the apparatus were the same as those described in Experiment 3.

#### Stimuli and procedure

**Fig 7:**
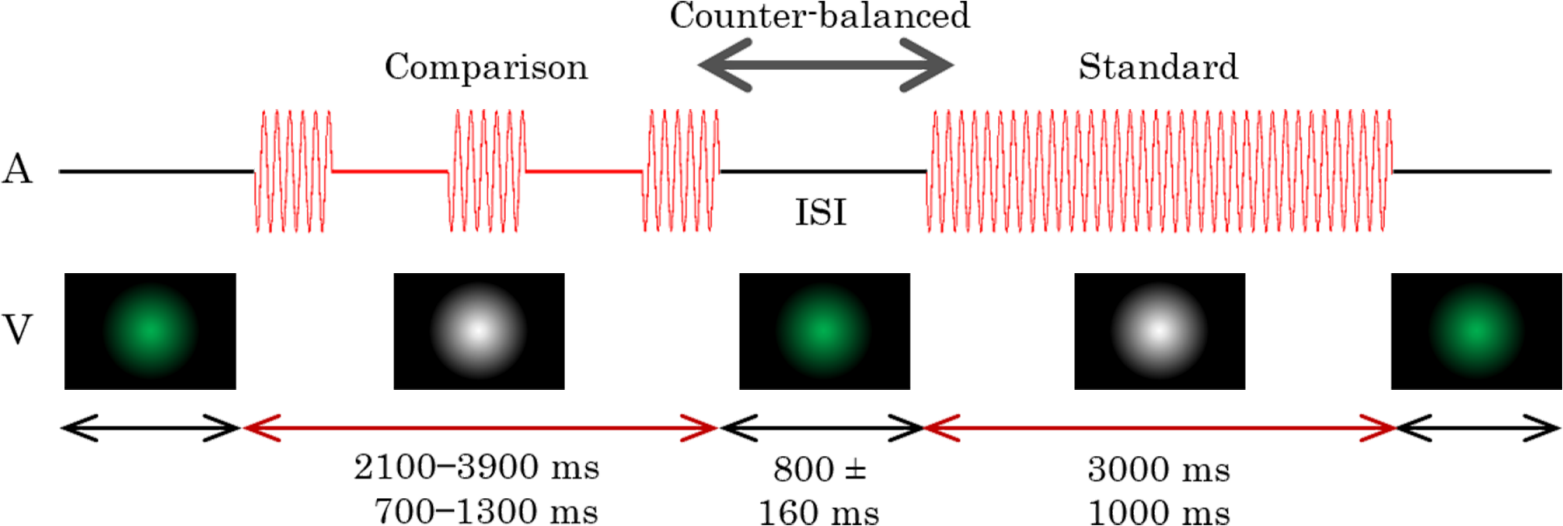
Time course of Experiment 4. Both auditory (A) and visual (V) stimuli were presented simultaneously. The visual stimuli were always presented continuously without flickering. The auditory comparison stimuli fluttered in half of the trials. The durations of comparison stimuli varied from those of the standard stimuli. The stimulus durations and ISI (inter-stimulus-interval) were varied in each trial.

Auditory and visual stimuli were presented simultaneously for the same duration. The standard stimuli were a continuously presented Gaussian blob (SD = 1.45°, 50.3 cd/m^2^) presented with a simple tone (1,000 Hz, 80 dB). The comparison stimuli were either the same as the standard stimuli or a continuously presented Gaussian blob presented with a 10.9-Hz auditory flutter. The stimulus configurations are shown in Fig 7.

The standard stimulus duration was either 3-s or 1-s, while the comparison stimulus duration was one of seven durations (different by 0%, ±10%, ±20%, or ±30% from the standard duration). Equiluminant green Gaussian blobs (SD = 1.45°) and silences were presented before the stimulus presentation, during the ISI, and after the stimulus presentation.

Subjects were instructed to attend to the visual stimuli while ignoring the auditory stimuli, and to indicate whether the second visual stimulus had a longer duration than the first by pressing a corresponding key. There was no time limit to respond. Participants were instructed not to estimate stimuli durations. Each condition (7 comparison durations × 2 types of comparison stimuli [continuous or flutter]) was repeated 40 times for each standard duration (3-s or 1-s), and each participant completed 1,120 trials across a 4-day period.

### Experiment 4: Results and Discussion

One participant was excluded from all analyses because his response proportions did not exceed chance, even when comparison durations were ±30% different from standard durations.

**Fig 8:**
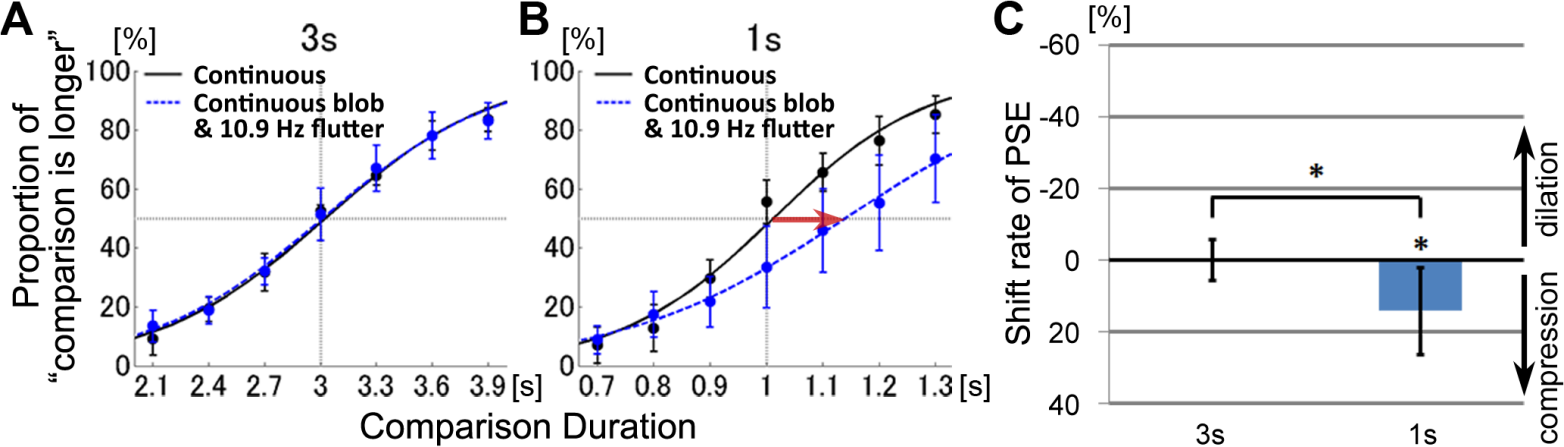
Experiment 4. Effects of auditory flutters on interval timing perception of visually presented stimulus. Psychometric functions showed the mean probability of the comparison stimulus perceived longer than the standard stimulus. The results for the standard duration of 3-s (A) and 1-s (B) were plotted separately. The red arrow indicates a significant PSE shift. (C) The PSE (point of subjective equality) shift rates between the conditions with continuous auditory tones and with the fluttering tones. The error bars represent the 95% confidence interval for each comparison duration.

The proportions that the comparison stimulus was perceived longer than the standard stimulus are plotted in Fig 8. Data were averaged across subjects and fitted to logistic psychometric functions. For the 3-s duration, the curves for the continuous stimuli and for the fluttering stimuli overlapped (Fig 8A), indicating that the auditory flutters did not induce distortions of the perceived duration for continuously presented visual stimulus for the 3-s condition. On the other hand, for the 1-s duration, the curve for the auditory flutters shifted to the right, indicating compression of the perceived duration (Fig 8B). The shift rates of the average PSEs were −0.80% ± 6.28% (SD) for the 3-s duration, and 14.17% ± 13.03% (SD) for the 1-s duration (Fig 8C). PSE shift rate was significantly different from zero for the 1-s duration (t(7) = 2.88, *p* = 0.03, *d* = 1.09), but not the 3-s duration (t(7) = 0.03, *p* = 0.97, *d* = 0.01). The shift rate difference between the 3-s and 1-s conditions was significant (t(7) = 3.08, *p* = 0.02).

These results indicate that perceived time was compressed by the 1-s auditory flutter, even when participants were told to ignore the auditory information. This compression was not observed with the 3-s duration. These results were similar to those reported for Experiment 1, where auditory flutters were presented and participants based their responses on auditory information. The perceived time compression observed in this experiment indicates that auditory flutters can affect interval timing perception of visually presented stimuli, and these effects remain even when the observer attempts to ignore the auditory stimulus.

## General Discussions

Our results indicate that peri-second auditory flutters induce time compression (Experiment 1), and that peri-second and supra-second visual flickers induce time dilation (Experiment 2). When auditory flutters and visual flickers were presented simultaneously for peri-second durations, their effects canceled out each other (Experiment 3). The time compression effect of auditory flutters remained, even when they were to be ignored (Experiment 4).

It should be emphasized that our results do not indicate that auditory flutters always induce time compression, or that visual flickers always induce time dilation. Rather, our results simply suggest that auditory flutters and visual flickers of the same duration and the same temporal frequency could differentially modulate interval timing perception. By examining interval timing perception in such conditions, we were able to gain insights into the modality-dependent mechanisms of interval timing perception, as well as the interactions between auditory and visual modalities.

### Modality effects in the internal clock model

It has been proposed that auditory and visual modalities depend on different internal clock systems, and that the basic clock of the pacemaker is faster for the auditory modality rather than the visual modality [6, 49–52, 73]. The internal clocks are associated with neural oscillations [4, 16, 17, 64, 65], and neuroimaging studies have shown the different contributions of auditory and visual oscillatory activities to temporal processing [74–76]. Kosem, Gramfort (75) reported that only auditory delta oscillations (1–2 Hz) successfully predicted the behavioral results during audio-visual simultaneity judgment, while both auditory and visual neural activities were entrained by repetitive presentation of audiovisual stimuli. It has also been reported that visual flickers entrain neural oscillations and change internal clock speeds, resulting in dilations of interval timing perception [55]. In the internal clock model, when the internal clock decelerates, the number of ticks per time decreases, thereby accumulating fewer ticks for a given duration. When the internal clock accelerates, the number of ticks per time increases, accumulating more ticks for a given duration. Therefore, time compression is attributed to a decelerated internal clock, while time dilation is attributed to an accelerated internal clock. In the present study, the auditory flutters of 10.9 Hz induced time compression and visual flickers of 10.9 Hz induced time dilation for peri-second interval timing perception. This implies that the auditory flutters of 10.9 Hz decelerated the internal clock, while the visual flickers of 10.9 Hz accelerated the internal clock in the peri-second measurements. Assuming both auditory flutters and visual flickers induce neural entrainments, this suggests that for the peri-second duration, the auditory pacemaker normally operates faster than 10.9 Hz, while the visual pacemaker normally operates slower than 10.9 Hz.

### Peri-second and supra-second durations

Several studies have suggested that interval timing perception for sub-second and supra-second durations relies on different neural mechanisms [2, 56–64]; however, there has been no consensus on what duration borders between sub- and supra-second interval timing perception [1, 56, 63, 64, 77]. In the present study, visual flickers induced time dilation with both peri-second and supra-second durations. However, auditory flutters did not induce any time distortion for the supra-second duration while they did induce time compression for the peri-second duration. The results suggest that time perception for the visual modality recruits similar mechanisms for peri-second and supra-second durations, while time perception for the auditory modality recruits different mechanisms for supra-second and peri-second durations. Generally, the auditory modality has higher temporal resolutions than the visual modality [34–36]. The auditory modality also shows a greater ability in discriminating shorter durations [34]. Therefore, it is plausible that the visual and auditory modalities differ in their mechanisms for peri- and supra-second durations. If we assume that the perception of shorter durations relies on automatic processing and that the perception of longer durations relies on cognitive processing [59, 60], the modality-dependent differences in the sub- and supra-second perceptions may be explained by the different boundaries for automatic processing and cognitive processing.

### Auditory-visual interactions

As we discussed above, visual and auditory modalities exhibited opposite effects (dilations and compressions, respectively) in the distortions of time perception. When flickering visual stimuli and fluttering auditory stimuli were presented simultaneously, the distortion of perceived time was reduced. These reduced effects indicate that the opposite effects of visual and auditory modalities can be integrated, and to some extent, be canceled out. With this simultaneous presentation, we observed a slight trend of time dilation with the supra-second duration, and a slight trend of time compression with the peri-second duration. These trends were consistent with the significant difference of PSE shift rates for the supra-second and peri-second duration. Many previous studies [43, 44, 78, 79] have reported auditory dominance in temporal processing, and our results were consistent with them. The dominance of the auditory modality was also observed in Experiment 4, where the visual stimulus was presented with auditory flutters, which the subjects were instructed to ignore. Despite instructions to ignore the auditory stimuli, the auditory flutters affected duration perception, inducing time compression. The trend of auditory dominance observed in this study appears to be inconsistent with the results reported by van Wassenhove, Buonomano (6). They showed that oddball visual stimulus distorts perceived duration of auditory stimulus, but oddball auditory stimulus does not distort the perceived duration of visual stimulus significantly, thereby suggesting the dominance of the visual modality. Chen and Yeh (43) followed this experiment and showed contradicting results and suggested that inequivalent magnitude of stimuli resulted in different dominance of the modalities for the time estimation tasks.

The reduced time distortions observed with the simultaneous audio-visual presentations can also be achieved by switching the dominant modality on a trial-by-trial basis. If the subjects chose which modality to attend to and switched their attention, the opposite effects could be averaged, yielding the seemingly canceled out time distortion effects. We cannot exclude this possibility from our data. However, our subjects reported that they perceived the simultaneously presented stimuli as integrated ones. In addition, as the subjects had difficulty ignoring the auditory information even though they were instructed to do so, it is unlikely that attention to the auditory and visual stimuli were switched on a trial-by-trial basis.

### Memory

Earlier in the general discussion, we interpreted our results within the framework of the pacemaker model, and proposed that the 10.9-Hz auditory flutters decelerated the internal pacemaker, while the 10.9-Hz visual flickers accelerated the internal pacemaker. Therefore, the decelerated pacemaker contributed to time compression and the accelerated pacemaker contributed to time dilation. However, inconsistent with our theory, Treisman, Faulkner (11) reported that auditory flutters of 11 Hz induced time dilation, using a verbal estimation task where participants reported estimated durations by typing on a keyboard. This inconsistency can be attributed to differences in the experimental tasks [69–72]. Such task dependency can be explained by the three-process model in the scalar timing theory.

Neural processing for time perception is classified into three stages: clock, memory, and decision [2, 4, 8, 47, 50, 80]. Perceived time is coded into a neural representation (clock stage), temporal information is stored in working memory (memory stage), and stored information is then compared to other temporal representations, so that the subjective time is obtained (decision stage). The pacemaker theory assumes that temporal frequencies of external stimuli, such as auditory flutters and visual flickers, entrain the base clocks of the internal pacemakers in the clock stage [12–16, 55]. On the other hand, it is known that stored and retrieved temporal information in the memory stage can also be affected by modulations of the external stimulus [13]. When interpreted using this framework, the first stimulus in our discrimination task was stored in reference memory, and the second stimulus was stored in working memory while being compared with the first stimulus. In contrast, the verbal estimation task used in Treisman, Faulkner (11) required the target stimuli to be stored in working memory, and compared to existing internal templates of the durations. In the three-process model, the distortion effect in the retrieval phase was opposite compared to the effect in the encoding phase, because the encoded duration was re-interpreted based on the altered base clocks of the pacemakers in the memory stage. In the discrimination task used in our study, the presentation orders of the standard and comparison stimuli were counterbalanced across the trials. In contrast, in the verbal estimation task, the encoded information was always compared with the existing internal templates, which could potentially introduce some effects in the decision stages.

If the memory stages affect time distortion differently, order of stimulus presentation may also affect the perceived time. In fact, preceding duration can be modulated during memory retention, thereby resulting in a bias in the discrimination thresholds and in sensitivity (69, 81–83). In addition, Ono and Kitazawa showed that the order of fluttering stimuli has an opposite effect on time distortion; flutters presented after a test stimulus compressed the perceived duration of the test stimulus, while flutters presented before a test stimulus dilated the perceived duration of the test stimulus. If the opposite effect existed between the presentation order of the standard and comparison stimuli, the effect could have been cancelled out by counter balancing. To examine the effect of the presentation order, we conducted within-subjects two-way ANOVA (2 types of comparison stimuli [continuous or flutter/flicker] × 2 presentation orders [the comparison stimulus presented first or the comparison stimulus presented second]) for the PSE shift of each experiment. The significant main effect of the presentation order was observed in the 1-s condition in Experiment 3 and 1-s and 3-s durations in Experiment 4 (F(8,1) = 6.92, p < 0.05; F(6,1) = 10.65, p < 0.05; F(6,1) = 8.89, p < 0.05, respectively). The interaction of the stimulus type (continuous or flutter/flicker) and the stimulus order (comparison first or standard first) was significant in the 3-s duration of Experiments 2 and 3 (F(8,1) = 5.92, p < 0.05; F(8,1) = 23.09, p < 0.01, respectively). The main effect of the presentation order and the interaction between the stimulus type and stimulus order were not significant in other experiments, indicating that the effect of the presentation order on the time dilation was marginal. Further investigation is required to ascertain how memory stage may affect the perceived time and how the effect of order is related to between-modality interactions.

## Conclusions

With the same temporal modulation of 10.9 Hz, visual flickers and auditory flutters induced opposite distortions of perceived duration. The distortions induced by auditory flutters were different for peri-second and supra-second durations. These results suggest that stimulus durations are coded and processed in modality-dependent networks, and that peri-second and supra-second durations can be modulated differently by externally induced entrainments.
